# Supplementary material for: Patterns of peripartum depression and anxiety during the pre-vaccine COVID-19 pandemic
Source: BMC Pregnancy Childbirth. 2024 Apr 25;24:310. doi: 10.1186/s12884-024-06518-8 (PMC11044399; doi:10.1186/s12884-024-06518-8)
Supplement: Supplementary file 1 — Supplementary Table 1A [file 12884_2024_6518_MOESM1_ESM.docx]

| Characteristics | 34 week visit, n = 298 | | | 6 week postpartum visit, n = 309 | | | 6 month postpartum visit, n = 243 | | |
| --- | --- | --- | --- | --- | --- | --- | --- | --- | --- |
|  | GAD >/=10 | OR (95% CI) | P-value | GAD >/=10 | OR (95% CI) | P-value | GAD >/=10 | OR (95% CI) | P-value |
| Age (year) |  | 1.01 (0.94-1.07) | 0.89 |  | 0.95 (0.88-1.02) | 0.17 |  | 0.94 (0.87-1.00) | 0.06 |
| GA at enrollment (week) |  | 0.97 (0.92-1.03) | 0.33 |  | 0.95 (0.89-1.02) | 0.13 |  | 0.99 (0.93-1.05) | 0.75 |
| GA at delivery (week) |  | 0.88 (0.75-1.02) | 0.10 |  | 1.07 (0.88-1.29) | 0.50 |  | 0.99 (0.87-1.12) | 0.82 |
| Nulliparous | 21 (17.1%) | 1.30 (0.69-2.40) | 0.43 | 15 (11.8%) | 1.39 (0.66-2.90) | 0.39 | 20 (20.4%) | 1.43 (0.74-2.80) | 0.29 |
| IVF pregnancy | 2 (6.3%) | 0.35 (0.08-1.50) | 0.16 | 2 (6.3%) | 0.57 (0.13-2.50) | 0.46 | 3 (13.0%) | 0.70 (0.20-2.50) | 0.57 |
| *Body mass index (kg/m^2^)* |  |  | 0.57 |  |  | 0.80 |  |  | 0.68 |
| Less than 25 | 17 (12.9%) | 1.00 |  | 13 (9.4%) | 1.00 |  | 22 (19.5%) | 1.00 |  |
| 25-30 | 16 (17.8%) | 1.46 (0.70-3.10) | 0.32 | 11 (12.0%) | 1.31 (0.56-3.10) | 0.54 | 11 (16.7%) | 0.83 (0.37-1.84) | 0.64 |
| 30 or higher | 12 (16.7%) | 1.35 (0.61-3.00) | 0.46 | 7 (9.5%) | 1.01 (0.38-2.60) | 0.99 | 9 (14.3%) | 0.69 (0.30-1.61) | 0.39 |
| *Race* |  |  | 0.97 |  |  | 0.17 |  |  | 0.93 |
| Asian | 3 (14.3%) | 0.92 (0.25-3.30) | 0.89 | 2 (10.0%) | 1.40 (0.29-6.70) | 0.67 | 0 (0.0%) | <0.001 |  |
| Black | 4 (20.0%) | 1.38 (0.43-4.40) | 0.59 | 1 (4.5%) | 0.60 (0.08-4.80) | 0.63 | 2 (12.5%) | 0.64 (0.14-3.00) | 0.57 |
| Hispanic/Latinx | 11 (14.1%) | 0.90 (0.42-1.94) | 0.79 | 14 (17.3%) | 2.60 (1.18-5.90) | 0.02* | 10 (18.9%) | 1.04 (0.47-2.30) | 0.93 |
| White | 26 (15.4%) | 1.00 |  | 13 (7.3%) | 1.00 |  | 28 (18.3%) | 1.00 |  |
| Other | 1 (11.1%) | 0.69 (0.08-5.70) | 0.73 | 1 (12.5%) | 1.80 (0.21-15.8) | 0.59 | 2 (28.6%) | 1.79 (0.33-9.70) | 0.50 |
| *Region of country* |  |  | 0.82 |  |  | 0.79 |  |  | 0.48 |
| Midwest | 7 (14.6%) | 1.00 |  | 3 (5.7%) | 1.00 |  | 4 (9.8%) | 1.00 |  |
| Northeast | 14 (18.7%) | 1.34 (0.50-3.60) | 0.56 | 8 (10.3%) | 1.91 (0.48-7.50) | 0.36 | 14 (21.9%) | 2.60 (0.79-8.50) | 0.12 |
| South | 11 (14.7%) | 1.01 (0.36-2.80) | 0.99 | 8 (10.5%) | 1.96 (0.50-7.80) | 0.34 | 11 (18.6%) | 2.10 (0.63-7.20) | 0.23 |
| West | 12 (13.5%) | 0.91 (0.33-2.50) | 0.86 | 9 (9.9%) | 1.83 (0.47-7.10) | 0.38 | 13 (17.8%) | 2.00 (0.61-6.60) | 0.25 |
| *Language* |  |  | 0.95 |  |  | 0.97 |  |  |  |
| English | 43 (15.4%) | 1.00 |  | 29 (10.0%) | 1.00 |  | 42 (17.6%) | 1.00 |  |
| Spanish | 2 (12.5%) | 0.78 (0.17-3.60) | 0.75 | 2 (11.8%) | 1.20 (0.26-5.50) | 0.82 | 0 (0.0%) | <0.001 |  |
| Heterosexual/Straight | 41 (14.3%) | 0.29 (0.08-1.04) | 0.06 | 28 (9.4%) | 0.31 (0.08-1.22) | 0.09 | 39 (16.8%) | 0.54 (0.14-2.10) | 0.38 |
| In a significant relationship | 44 (15.7%) | 2.8 (0.36-21.6) | 0.33 | 30 (10.3%) | 1.84 (0.24-14.4) | 0.56 | 38 (16.7%) | 0.55 (0.17-1.83) | 0.33 |
| *Employment status* |  |  | 0.54 |  |  | 0.72 |  |  | 0.03* |
| Full time, paid | 35 (16.9%) | 1.00 |  | 24 (11.0%) | 1.00 |  | 29 (17.2%) | 1.00 |  |
| Full time homemaker/childcare | 2 (7.7%) | 0.41 (0.09-1.81) | 0.24 | 1 (3.8%) | 0.33 (0.04-2.50) | 0.28 | 2 (9.5%) | 0.51 (0.11-2.30) | 0.38 |
| Part time paid/Seeking | 4 (10.5%) | 0.58 (0.19-1.73) | 0.33 | 3 (8.3%) | 0.74 (0.21-2.60) | 0.64 | 3 (9.1%) | 0.48 (0.14-1.69) | 0.25 |
| Other | 4 (14.8%) | 0.86 (0.28-2.60) | 0.78 | 3 (10.7%) | 0.98 (0.27-3.50) | 0.97 | 8 (40.0%) | 3.20 (1.21-8.60) | 0.02* |
| *Annual income ($)* |  |  | 0.56 |  |  | 0.40 |  |  | 0.04* |
| Less than 25,000 | 1 (4.2%) | 1.00 |  | 2 (8.7%) | 1.00 |  | 1 (5.6%) | 1.00 |  |
| 25,000-50,000 | 7 (15.9%) | 4.40 (0.50-37.7) | 0.18 | 7 (15.9%) | 1.99 (0.38-10.5) | 0.42 | 11 (32.4%) | 8.10 (0.96-69.2) | 0.06 |
| 50,000-100,000 | 12 (15.0%) | 4.10 (0.50-32.9) | 0.19 | 10 (11.6%) | 1.38 (0.28-6.80) | 0.69 | 13 (20.0%) | 4.20 (0.52-34.9) | 0.18 |
| More than 100,000 | 23 (16.3%) | 4.50 (0.58-34.9) | 0.15 | 11 (7.5%) | 0.85 (0.18-4.10) | 0.84 | 16 (13.2%) | 2.60 (0.32-20.8) | 0.37 |
| Tobacco, alcohol, and/or marijuana use | 1 (7.7%) | 0.46 (0.06-3.60) | 0.46 | 2 (12.5%) | 1.30 (0.28-6.00) | 0.74 | 4 (36.4%) | 2.90 (0.81-10.5) | 0.10 |
| Healthcare worker | 16 (17.4%) | 1.73 (0.59-5.10) | 0.32 | 9 (9.3%) | 0.66 (0.23-1.88) | 0.43 | 12 (14.6%) | 0.78 (0.28-2.20) | 0.64 |
| *Medical history* |  |  | <.0001** |  |  | 0.0004** |  |  | 0.001** |
| No pre-existing conditions | 18 (11.4%) | 1.00 |  | 7 (4.3%) | 1.00 |  | 16 (13.1%) | 1.00 |  |
| Medical co-morbidities | 2 (3.5%) | 0.28 (0.06-1.26) | 0.10 | 6 (9.7%) | 2.40 (0.77-7.40) | 0.13 | 4 (8.0%) | 0.58 (0.18-1.82) | 0.35 |
| Mental health co-morbidities | 25 (31.3%) | 3.50 (1.79-7.00) | 0.0003** | 18 (22.0%) | 6.20 (2.50-15.6) | <.0001** | 22(32.4%) | 3.20 (1.53-6.60) | 0.002** |
| *Antepartum complications* |  |  | 0.003** |  |  | 0.30 |  |  | 0.31 |
| None | 16 (9.0%) | 1.00 |  | 15 (8.1%) | 1.00 |  | 25 (17.5%) | 1.00 |  |
| Gestational diabetes | 7 (19.4%) | 4.40 (1.58-12.3) | 0.005** | 4 (19.0%) | 2.70 (0.80-8.90) | 0.11 | 3 (17.6%) | 1.01 (0.27-3.80) | 0.99 |
| Hypertensive disease of pregnancy | 8 (21.1%) | 2.70 (1.06-6.80) | 0.04* | 4 (10.8%) | 1.37 (0.43-4.40) | 0.59 | 4 (12.5%) | 0.67 (0.22-2.10) | 0.50 |
| Oligo/Polyhydramnios or PPROM | 4 (15.4%) | 1.83 (0.56-6.00) | 0.32 | 2 (7.7%) | 0.94 (0.20-4.40) | 0.94 | 2 (9.5%) | 0.50 (0.11-2.30) | 0.37 |
| Other | 10 (33.3%) | 5.00 (2.00-12.6) | 0.006** | 6 (18.2%) | 2.50 (0.90-7.10) | 0.08 | 8 (32.0%) | 2.20 (0.86-5.70) | 0.10 |
| Intrapartum complications | 13 (23.2%) | 1.89 (0.92-3.90) | 0.09 | 6 (10.2%) | 0.96 (0.38-2.50) | 0.93 | 10 (18.9%) | 1.08 (0.49-2.40) | 0.86 |
| NICU admission | 7 (19.4%) | 1.42 (0.58-3.50) | 0.44 | 4 (10.0%) | 1.00 (0.33-3.00) | 0.99 | 5 (16.1%) | 0.91 (0.33-2.50) | 0.86 |
| Infant ”roomed in” after delivery | 37 (14.5%) | 0.74 (0.32-1.73) | 0.49 | 28 (10.7%) | 1.76 (0.51-6.00) | 0.37 | 39 (18.9%) | 2.60 (0.77-9.10) | 0.12 |
| Hospitalized at enrollment | 0 (0.0%) | <0.001 |  | 0 (0.0%) | <0.001 |  | 0 (0.0%) | <0.001 |  |
| Quarantined at enrollment | 31 (17.2%) | 1.55 (0.78-3.00) | 0.21 | 24 (12.6%) | 2.30 (0.96-5.60) | 0.06 | 26 (17.7%) | 1.07 (0.54-2.10) | 0.84 |
| *Currently…* |  |  |  |  |  |  |  |  |  |
| COVID+ | 20 (13.0%) | 0.71 (0.38-1.34) | 0.29 | 11 (7.1%) | 0.50 (0.23-1.09) | 0.08 | 23 (19.0%) | 1.27 (0.65-2.50) | 0.48 |
| Hospitalized | 0 (0.0%) | <0.001 |  | 0 (0.0%) | <0.001 |  | 0 (0.0%) | <0.001 |  |
| Quarantined | 10 (27.8%) | 2.50 (1.11-5.60) | 0.03* | 6 (16.2%) | 1.91 (0.73-5.00) | 0.19 | 5 (15.6%) | 0.87 (0.32-2.40) | 0.79 |
| EPDS score at enrollment |  | 1.31 (1.16-1.47) | <.0001** |  | 1.17 (1.04-1.31) | 0.01* |  | 1.14 (1.02-1.27) | 0.02* |
| GAD-7 score at enrollment |  | 1.39 (1.21-1.58) | <.0001** |  | 1.23 (1.08-1.40) | 0.002** |  | 1.17 (1.04-1.32) | 0.01* |

**Supplemental Table 1A.** Univariable logistic regression analysis of demographic and clinical risk factors for symptomatic depression (EPDS >/=13 & suicidality) antepartum and postpartum. *p<0.05, **p<0.01
