# Supplementary material for: Patterns of peripartum depression and anxiety during the pre-vaccine COVID-19 pandemic
Source: BMC Pregnancy Childbirth. 2024 Apr 25;24:310. doi: 10.1186/s12884-024-06518-8 (PMC11044399; doi:10.1186/s12884-024-06518-8)
Supplement: Supplementary file 3 — Supplementary Table 2A [file 12884_2024_6518_MOESM3_ESM.docx]

**Supplemental Table 2A:** Table displays multivariate logistic regression analyses measuring the association of various demographic and clinical characteristics with symptomatic depression (EPDS >/=13) in the antepartum and postpartum. Each model was adjusted for age, parity, region of country, employment status, relationship status, and annual income. *p<0.05, **p<0.01

| *Characteristics* | *34 week visit, n = 279* | | | *6 week postpartum visit, n = 285* | | | *6 month postpartum visit, n = 230* | | |
| --- | --- | --- | --- | --- | --- | --- | --- | --- | --- |
|  | *EPDS >/=13* | *OR (95% CI)* | *P-value* | *EPDS >/=13* | *OR (95% CI)* | *P-value* | *EPDS >/=13* | *OR (95% CI)* | *P-value* |
| GA at enrollment (week) |  | 0.99 (0.93-1.06) | 0.80 |  | 0.95 (0.88-1.03) | 0.20 |  | 1.01 (0.95-1.08) | 0.67 |
| GA at delivery (week) |  | 0.92 (0.77-1.10) | 0.40 |  | 0.89 (0.79-1.00) | 0.05 |  | 0.98 (0.86-.11) | 0.74 |
| IVF pregnancy | 1 (3.3%) | 0.17 (0.02-1.33) | 0.09 | 2 (6.7%) | 0.93 (0.19-4.50) | 0.93 | 2 (9.1%) | 0.30 (0.06-.52) | 0.15 |
| *Body mass index (kg/m^2^)* |  |  | 0.52 |  |  | 0.47 |  |  | 0.99 |
| Less than 25 | 15 (12.0%) | 1.00 |  | 10 (7.7%) | 1.00 |  | 21 (19.4%) | 1.00 |  |
| 25-30 | 14 (16.9%) | 1.61 (0.70-3.70) | 0.26 | 11 (12.9%) | 1.75 (0.67-4.60) | 0.25 | 14 (23.0%) | 1.04 (0.45-2.40) | 0.93 |
| 30 or higher | 10 (14.7%) | 1.13 (0.45-2.90) | 0.80 | 7 (10.0%) | 1.07 (0.36-3.20) | 0.90 | 13 (21.7%) | 0.97 (0.40-2.30) | 0.94 |
| *Race* |  |  | 0.94 |  |  | 0.81 |  |  | 0.65 |
| White | 24 (14.8%) | 1.00 |  | 17 (10.0%) | 1.00 |  | 33 (22.6%) | 1.00 |  |
| Hispanic/Latinx | 10 (14.1%) | 0.86 (0.34-2.20) | 0.76 | 7 (9.9%) | 0.59 (0.20-1.79) | 0.35 | 8 (16.3%) | 0.56 (0.21-1.47) | 0.24 |
| Asian | 2 (11.8%) | 0.82 (0.17-4.00) | 0.80 | 1 (5.9%) | 0.48 (0.06-4.20) | 0.50 | 1 (8.3%) | 0.38 (0.04-3.20) | 0.37 |
| Black | 2 (10.0%) | 0.47 (0.09-2.50) | 0.38 | 2 (9.1%) | 0.46 (0.08-2.60) | 0.38 | 4 (25.0%) | 0.54 (0.14-2.20) | 0.38 |
| Other | 1 (12.5%) | 0.84 (0.08-8.40) | 0.88 | 1 (14.3%) | 0.94 (0.09-9.70) | 0.96 | 2 (33.3%) | 0.94 (0.14-6.50) | 0.95 |
| *Language* |  |  | 1.00 |  |  | 1.00 |  |  |  |
| English | 39 (14.8%) | 1.00 |  | 28 (10.3%) | 1.00 |  | 48 (21.3%) | 1.00 |  |
| Spanish | 0 (0.0%) | <0.001 |  | 0 (0.0%) | <0.001 |  | 0 (0.0%) | <0.001 |  |
| Other | 0 (0.0%) | <0.001 |  | 0 (0.0%) | <0.001 |  | 0 (0.0%) | <0.001 |  |
| Heterosexual/Straight | 36 (13.3%) | 0.21 (0.05-1.01) | 0.05 | 25 (9.0%) | 0.29 (0.06-1.42) | 0.13 | 45 (20.8%) | 1.11 (0.8-6.70) | 0.91 |
| Tobacco, alcohol, and/or marijuana use | 1 (9.1%) | 0.81 (0.10-7.00) | 0.85 | 3 (21.4%) | 2.80 (0.67-11.9) | 0.16 | 4 (36.4%) | 2.00 (0.49-8.20) | 0.34 |
| Healthcare worker | 17 (19.3%) | 1.69 (0.52-5.50) | 0.38 | 9 (9.7%) | 2.8 (0.53-15.2) | 0.23 | 16 (20.5%) | 0.93 (0.32-2.70) | 0.9 |
| *Medical history* |  |  | <.0001** |  |  | <.0001** |  |  | 0.02* |
| No pre-existing conditions | 12 (8.0%) | 1.00 |  | 7 (4.6%) | 1.00 |  | 21 (17.8%) | 1.00 |  |
| Medical co-morbidities | 4 (7.7%) | 0.83 (0.25-2.80) | 0.76 | 3 (5.4%) | 1.38 (0.33-5.90) | 0.66 | 6 (13.3%) | 0.56 (0.20-1.62) | 0.29 |
| Mental health co-morbidities | 23 (30.7%) | 6.80 (2.90-15.7) | <.0001** | 18 (23.4%) | 8.5 (3.10-23.1) | <.0001** | 21 (32.3%) | 2.4 (1.08-5.2) | 0.03* |
| *Antepartum complications* |  |  | 0.08 |  |  | 0.39 |  |  | 0.26 |
| Gestational diabetes | 4 (19.0%) | 2.4 (0.66-8.60) | 0.18 | 3 (14.3%) | 1.46 (0.35-6.10) | 0.60 | 2 (13.3%) | 0.39 (0.07-2.20) | 0.28 |
| Hypertensive disease of pregnancy | 9 (24.3%) | 3.2 (1.13-8.90) | 0.03* | 2 (5.6%) | 0.60 (0.12-2.90) | 0.53 | 7 (22.6%) | 1.00 (0.36-2.80) | 0.99 |
| Oligo/Polyhydramnios or PPROM | 2 (8.0%) | 0.94 (0.20-4.50) | 0.94 | 1 (4.0%) | 0.52 (0.06-4.30) | 0.54 | 5 (25.0%) | 1.81 (0.56-5.80) | 0.32 |
| Other | 6 (23.%) | 3.5 (1.1-11.0) | 0.03* | 5 (17.2%) | 2.80 (0.80-9.50) | 0.11 | 9 (37.5%) | 2.5 (0.89-7.30) | 0.08 |
| Intrapartum complications | 9 (16.7%) | 1.31 (0.54-3.20) | 0.55 | 6 (10.7%) | 1.47 (0.52-4.20) | 0.47 | 13 (25.5%) | 1.42 (0.63-3.20) | 0.40 |
| NICU admission | 7 (20.0%) | 1.83 (0.69-4.90) | 0.23 | 6 (15.8%) | 2.10 (0.76-6.00) | 0.15 | 9 (30.0%) | 1.70 (0.66-4.40) | 0.27 |
| Infant ”roomed in” after delivery | 34 (14.2%) | 1.1 (0.39-3.20) | 0.85 | 23 (9.4%) | 0.75 (0.25-2.20) | 0.59 | 43 (22.1%) | 1.89 (0.62-5.80) | 0.26 |
| Hospitalized at enrollment | 0 (0.0%) | <0.001 |  | 0 (0.0%) | <0.001 |  | 0 (0.0%) | <0.001 |  |
| Quarantined at enrollment | 29 (17.4%) | 2.20 (1.00-5.00) | 0.05 | 19 (10.9%) | 1.2 (0.49-2.80) | 0.72 | 29 (21.0%) | 0.93 (0.46-1.89) | 0.85 |
| *Currently…* |  |  |  |  |  |  |  |  |  |
| COVID+ | 15 (10.4%) | 0.51 (0.25-1.06) | 0.07 | 12 (8.2%) | 0.59 (0.26-1.35) | 0.21 | 25 (21.9%) | 0.99 (0.50-1.94) | 0.97 |
| Hospitalized | 0 (0.0%) | <0.001 |  | 0 (0.0%) | <0.001 |  | 0 (0.0%) | <0.001 |  |
| Quarantined | 6 (18.2%) | 1.53 (0.55-4.30) | 0.42 | 5 (15.2%) | 1.81 (0.57-5.80) | 0.32 | 6 (20.0%) | 0.75 (0.26-2.20) | 0.60 |
| EPDS score at enrollment |  | 1.58 (1.19-2.10) | 0.002** |  | 1.8 (1.00-1.39) | 0.05 |  | 1.16 (1.00-1.36) | 0.06 |
| GAD-7 score at enrollment |  | 1.39 (1.4-1.69) | 0.001** |  | 1.21 (1.02-1.44) | 0.03* |  | 1.19 (1.00-1.43) | 0.06 |
