# Supplementary material for: Patterns of peripartum depression and anxiety during the pre-vaccine COVID-19 pandemic
Source: BMC Pregnancy Childbirth. 2024 Apr 25;24:310. doi: 10.1186/s12884-024-06518-8 (PMC11044399; doi:10.1186/s12884-024-06518-8)
Supplement: Supplementary file 4 — Supplementary Table 2B [file 12884_2024_6518_MOESM4_ESM.docx]

**Supplemental Table 2B:** Table displays multivariate logistic regression analyses measuring the association of various demographic and clinical characteristics with symptomatic anxiety (GAD>/=10) in the antepartum and postpartum. Each model was adjusted for age, parity, region of country, employment status, relationship status, and annual income. *p<0.05, **p<0.01

| *Characteristics* | *34 week visit, n = 276* | | | *6 weeks postpartum visit, n = 287* | | | *6 months postpartum visit, n = 230* | | |
| --- | --- | --- | --- | --- | --- | --- | --- | --- | --- |
|  | *GAD >/=10* | *OR (95% CI)* | *P-value* | *GAD >/=10* | *OR (95% CI)* | *P-value* | *GAD >/=10* | *OR (95% CI)* | *P-value* |
| GA at enrollment (week) |  | 0.97 (0.91-1.03) | 0.27 |  | 0.94 (0.87-1.01) | 0.10 |  | 1.00 (0.93-1.07) | 0.86 |
| GA at delivery (week) |  | 0.91 (0.77-1.09) | 0.30 |  | 1.11 (0.87-1.39) | 0.42 |  | 1.01 (0.85-1.18) | 0.96 |
| IVF pregnancy | 2 (6.7%) | 0.29 (0.06-1.31) | 0.11 | 2 (6.7%) | 0.72 (0.15-3.50) | 0.69 | 3 (13.6%) | 0.71 (0.17-3.00) | 0.65 |
| *Body mass index (kg/m^2^)* |  |  | 0.73 |  |  | 0.66 |  |  | 0.18 |
| Less than 25 | 17 (13.7%) | 1.00 |  | 10 (7.7%) | 1.00 |  | 21 (19.4%) | 1.00 |  |
| 25-30 | 13 (15.9%) | 1.26 (0.56-2.80) | 0.58 | 10 (11.8%) | 1.55 (0.59- 4.10) | 0.37 | 10 (16.4%) | 0.46 (0.17-1.21) | 0.11 |
| 30 or higher | 11 (16.4%) | 1.40 (0.58-3.40) | 0.46 | 10 (11.8%) | 1.14 (0.38-3.40) | 0.81 | 9 (15.0%) | 0.44 (0.15-1.28) | 0.13 |
| *Race* |  |  | 0.94 |  |  | 0.46 |  |  | 0.71 |
| White | 25 (15.5%) | 1.00 |  | 13 (7.7%) | 1.0000 |  | 27 (18.5%) | 1.00 |  |
| Hispanic/Latinx | 9 (13.0%) | 1.06 (0.44-2.60) | 0.89 | 11 (15.5%) | 1.87 (0.70-5.00) | 0.21 | 9 (18.4%) | 0.91 (0.35-2.40) | 0.85 |
| Asian | 2 (11.8%) | 0.73 (0.15-3.50) | 0.69 | 1 (5.9%) | 0.59 (0.07-5.00) | 0.63 | 0 (0.0%) | <0.001 |  |
| Black | 4 (20.0%) | 1.64 (0.44-6.20) | 0.47 | 1 (4.5%) | 0.36 (0.04-3.40) | 0.37 | 2 (12.5%) | 0.31 (0.05-1.89) | 0.20 |
| Other | 1 (12.5%) | 1.01 (0.11-9.40) | 0.99 | 1 (14.3%) | 1.41 (0.13-15.0) | 0.78 | 2 (33.3%) | 1.78 (0.23-13.5) | 0.58 |
| *Language* |  |  | 1.00 |  |  | 0.90 |  |  |  |
| English | 41 (15.7%) | 1.00 |  | 26 (9.6%) | 1.00 |  | 40 (17.8%) | 1.00 |  |
| Spanish | 0 (0.0%) | <0.001 |  | 1 (8.3%) | 0.59 (0.06-6.00) | 0.65 | 0 (0.0%) | <0.001 |  |
| Other | 0 (0.0%) | <0.001 |  | 0 (0.0%) | <0.001 |  | 0 (0.0%) | <0.001 |  |
| Heterosexual/Straight | 37 (13.9%) | 0.12 ( 0.02-0.60) | 0.01* | 24 (8.7%) | 0.27 (0.06-1.30) | 0.10 | 37 (16.7%) | 0.54 (0.10-2.90) | 0.47 |
| Tobacco, alcohol, and/or marijuana use | 1 (9.1%) | 0.52 (0.06-4.30) | 0.55 | 1 (7.1%) | 0.56 (0.06-4.90) | 0.60 | 4 (36.4%) | 1.90 (0.45-8.00) | 0.38 |
| Healthcare worker | 16 (18.4%) | 2.70 (0.74-9.90) | 0.13 | 9 (9.8%) | 1.19 (0.33-4.20) | 0.79 | 12 (15.4%) | 1.07 (0.31-3.80) | 0.92 |
| *Medical history* |  |  | <.0001** |  |  | <.0001** |  |  | 0.0005** |
| No pre-existing conditions | 17 (11.4%) | 1.00 |  | 6 (3.9%) | 1.00 |  | 15 (12.7%) | 1.00 |  |
| Medical co-morbidities | 1 (2.0%) | 0.14 (0.02-1.08) | 0.06 | 5 (8.9%) | 2.60 (0.72-9.50) | 0.14 | 3 (6.7%) | 0.34 (0.08-1.38) | 0.16 |
| Mental health co-morbidities | 23 (31.1%) | 4.00 (1.88-8.60) | 0.0003** | 16 (20.8%) | 10.4 (3.50-31.0) | <.0001** | 22 (33.8%) | 3.70 (1.59-8.8) | 0.002** |
| *Antepartum complications* |  |  | 0.034* |  |  | 0.13 |  |  | 0.75 |
| Gestational diabetes | 6 (28.6%) | 4.00 (1.26-12.7) | 0.02* | 4 (20.0%) | 3.10 (0.81-11.9) | 0.1 | 3 (20.0%) | 0.74 (1.7-3.30) | 0.69 |
| Hypertensive disease of pregnancy | 8 (21.6%) | 3.20 (1.13-9.00) | 0.03* | 4 (11.1%) | 2.00 (0.55-7.50) | 0.28 | 4 (12.9%) | 0.50 (0.14-1.80) | 0.29 |
| Oligo/Polyhydramnios or PPROM | 4 (16.0%) | 2.00 (0.59-6.90) | 0.26 | 2 (8.0%) | 1.63 (0.32-8.20) | 0.56 | 2 (10.0%) | 0.56 (0.12-2.80) | 0.48 |
| Other | 8 (30.8%) | 3.70 (1.31-10.6) | 0.01* | 6 (20.7%) | 4.60 (1.37-15.7) | 0.01* | 7 (29.2%) | 1.23 (0.40-3.80) | 0.72 |
| Intrapartum complications | 12 (22.6%) | 1.66 (0.75-3.70) | 0.21 | 6 (10.7%) | 1.38 (0.49-3.90) | 0.54 | 10 (19.6%) | 1.39 (0.57-3.40) | 0.47 |
| NICU admission | 6 (17.6%) | 1.47 (0.54-4.00) | 0.45 | 4 (10.5%) | 1.24 (0.38-4.00) | 0.72 | 5 (16.7%) | 0.80 (0.24-2.60) | 0.71 |
| Infant ”roomed in” after delivery | 35 (14.8%) | 0.80 (0.30-2.10) | 0.65 | 24 (9.8%) | 1.45 (0.40-5.30) | 0.57 | 37 (19.0%) | 4.40 (0.95-19.9) | 0.06 |
| Hospitalized at enrollment | 0 (0.0%) | <0.001 |  | 0 (0.0%) | <0.001 |  | 0 (0.0%) | <0.001 |  |
| Quarantined at enrollment | 28 (17.1%) | 1.68 (0.81-3.50) | 0.16 | 22 (12.6%) | 3.10 (1.08-8.70) | 0.04* | 25 (18.1%) | 0.96 (0.44-2.10) | 0.93 |
| *Currently…* |  |  |  |  |  |  |  |  |  |
| COVID+ | 16 (11.1%) | 0.52 (0.26-1.06) | 0.07 | 10 (6.8%) | 0.45 (0.19-1.07) | 0.07 | 22(19.3%) | 1.24 (0.59-2.60) | 0.57 |
| Hospitalized | 0 (0.0%) | <0.001 |  | 0 (0.0%) | <0.001 |  | 0 (0.0%) | <0.001 |  |
| Quarantined | 8 (25.0%) | 2.70 (1.03-7.10) | 0.04* | 6 (18.2%) | 3.50 (1.14-10.6) | 0.03* | 5 (16.7%) | 0.74 (0.23-2.30) | 0.60 |
| EPDS score at enrollment |  | 1.51 (1.23-1.85) | <.0001** |  | 1.21 (0.99-1.47) | 0.07 |  | 1.28 (1.03-1.59) | 0.02* |
| GAD-7 score at enrollment |  | 1.75 (1.34-2.30) | <.0001** |  | 1.28 (1.04-1.56) | 0.018* |  | 1.47 (1.13-1.93) | 0.005** |
